# Supplementary material for: Domoic Acid and Pseudo-nitzschia spp. Connected to Coastal Upwelling along Coastal Inhambane Province, Mozambique: A New Area of Concern
Source: Toxins (Basel). 2021 Dec 15;13(12):903. doi: 10.3390/toxins13120903 (PMC8704230; doi:10.3390/toxins13120903)
Supplement: Supplementary file 1 [file toxins-13-00903-s001.zip › toxins-1488271-supplementary.pdf]

Supplementary Materials: Domoic Acid and *Pseudo-nitzschia* spp. Connected to Coastal Upwelling along Coastal Inhambane Province, Mozambique: A New Area of Concern

Holly Kelchner, Katie E. Reeve-Arnold, Kathryn M. Schreiner, Sibel Bargu, Kim G. Roques and Reagan M. Errera

Table S1. Pigment: chlorophyll a starting and output ratios in the CHEMTAX analysis of HPLC pigments. Starting ratios derived from Barlow *et al*. [60] and Higgens *et al*. [119]. Chl a-chlorophyll a; Chl b-chlorophyll b; Chl c1-chlorophyll c_1; Chl c2-chlorophyll c_2; Chl c3-chlorophyll c_3; Per-perodinin; But-butanoyloxyfucoxanthin; Fuc-fucoxanthin; Neo-neoxanthin; Viol-violaxanthin; Pras-prasinoxanthin; Hex-19′-hexanoyloxyfucoxanthin; Allo-alloxanthin; Zea-zeaxanthin; Anth-antheraxanthin; Lut-lutein.

Group	Chl *a*	Chl *b*	Chl *c* 1+2	Chl *c* 3	Per	But	Fuc	Neo	Viol	Pras	Hex	Allo	Zea	Anth	Lut
**Starting Ratios**															
Diatoms-1 (ex: *chaetoceros*)	1	0	0.022	0	0	0	0.201	0	0	0	0	0	0	0	0
Diatoms-2 (*Pseudo-nitzschia*)	1	0	0.125	0.062	0	0	0.371	0	0	0	0	0	0	0	0
Dinoflagellates-1	1	0	0.124	0	0.315	0	0	0	0	0	0	0	0	0	0
Dinoflagellates-2	1	0	0.050	0.196	0	0.033	0.103	0	0	0	0.076	0	0	0	0
Cryptophytes	1	0	0.127	0	0	0	0	0	0	0	0	0.241	0	0	0
Pelagophytes	1	0	0.125	0.024	0	0.292	0.245	0	0	0	0	0	0	0	0
Prasinophytes	1	0.324	0	0	0	0	0	0.037	0.071	0	0	0	0.013	0.012	0.029
Chlorophytes	1	0.194	0	0	0	0	0	0.040	0.030	0	0	0	0.019	0.008	0.103
Cyanobacteria (*Trichodesmium*)	1	0	0	0	0	0	0	0	0	0	0	0	0.030	0	0
**Final Ratio**															
Diatoms-1 (ex: *chaetoceros*)	1	0	0.017	0	0	0	0.210	0	0	0	0	0	0	0	0
Diatoms-2 (*Pseudo-nitzschia*)	1	0	0.116	0.049	0	0	0.352	0	0	0	0	0	0	0	0
Dinoflagellates-1	1	0	0.086	0	0.219	0	0	0	0	0	0	0	0	0	0
Dinoflagellates-2	1	0	0.035	0.146	0	0.023	0.071	0	0	0	0.035	0	0	0	0
Cryptophytes	1	0	0.093	0	0	0	0	0	0	0	0	0.176	0	0	0
Pelagophytes	1	0	0.073	0.014	0	0.187	0.143	0	0	0	0	0	0	0	0
Prasinophytes	1	0.218	0	0	0	0	0	0.025	0.048	0	0	0	0.009	0.008	0.020
Chlorophytes	1	0.139	0	0	0	0	0	0.029	0.021	0	0	0	0.014	0.006	0.074
Cyanobacteria (*Trichodesmium*)	1	0	0	0	0	0	0	0	0	0	0	0	0.027	0	0

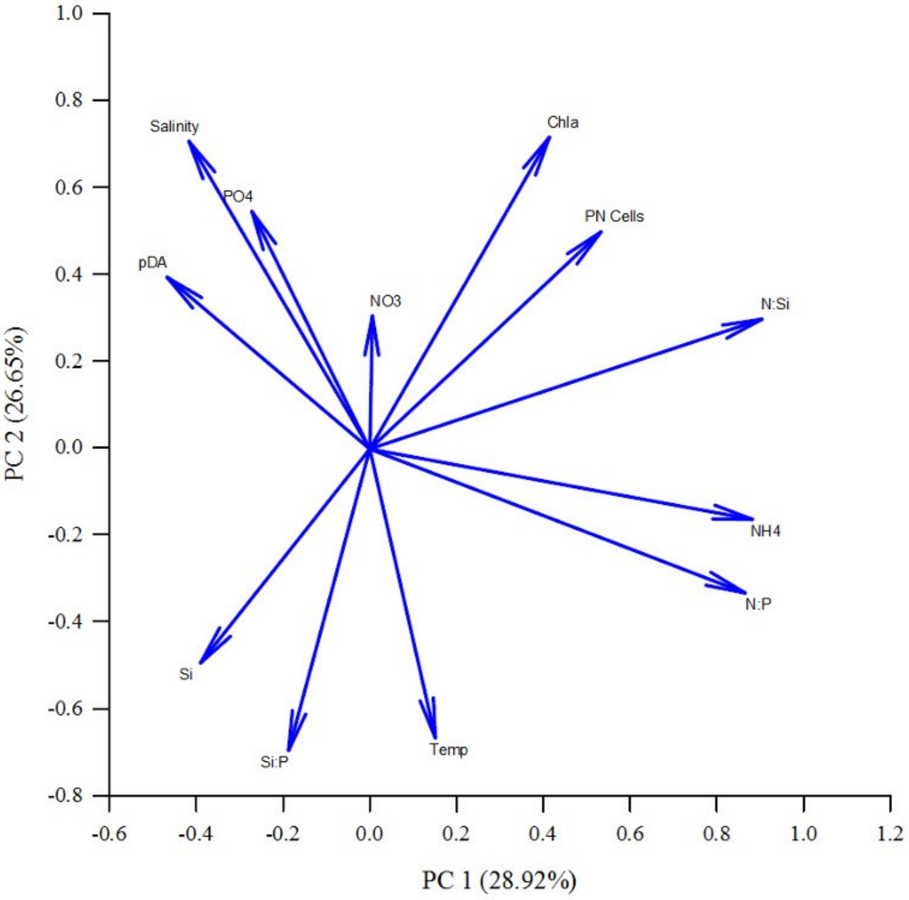

Figure S1. Principle Components Analysis. Variable factors from May to August 2018 defined by the two first axes showing the link between physiochemical parameters, chla, pDA and *Pseudo-nitzschia* spp. abundance.

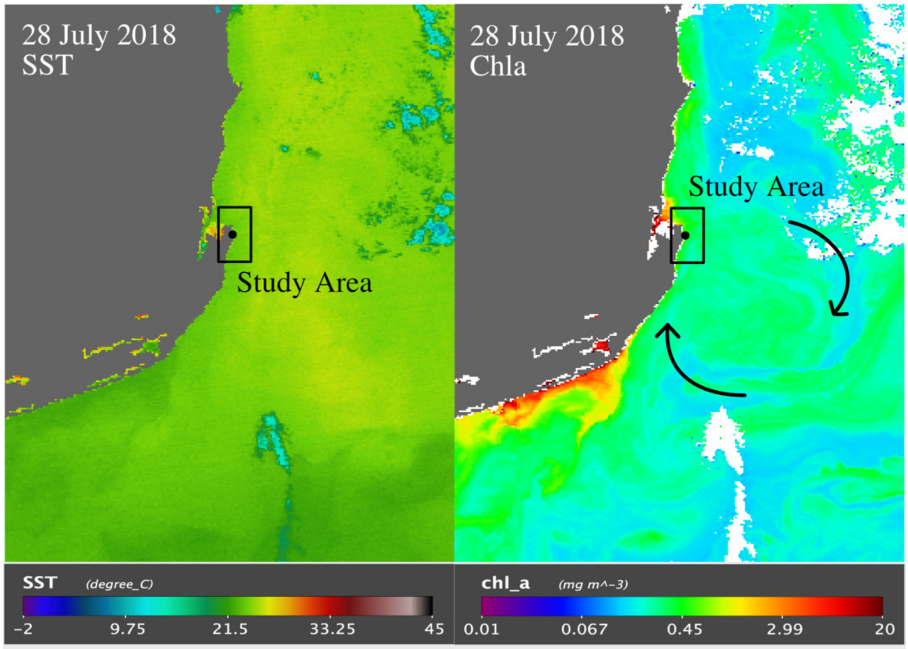

Figure S2. Satellite Imagery. Sea surface temperature (SST) and chlorophyll a (chla) images from 28 July 2018 at 13:15 LST reveal the occurrence of a cyclonic eddy along the coast of Inhambane Province. Estimate of chlorophyll a inferred from MODIS Aqua image produced using SeaDAS with NASA standard algorithms.

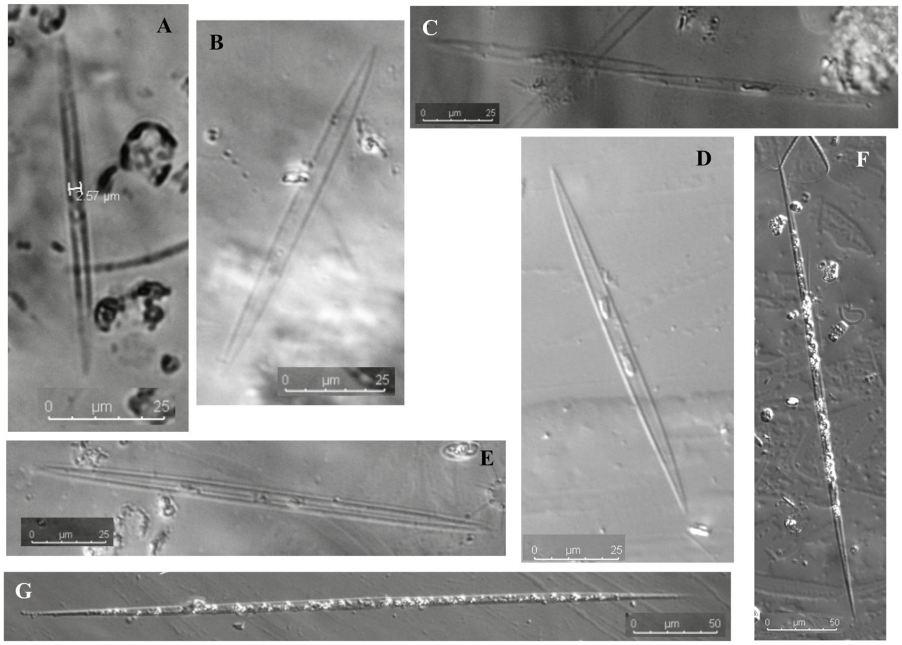

Figure S3. *Pseudo-nitzschia* spp. light microscopy analysis revealed at least three species of *Pseudo-nitzschia* spp. present in samples from coastal Inhambane Province based on width and shape. One type (**A**,**B**) had a width of 3 μm and a short lenth (>100 μm). Another type (**C**–**E**) had a similar length (>100 μm), but a width of 5μm. The third type (**F**,**G**) was distinguished by the very long cell shape (200+ μm) with finely pointed ends and a width of 5μm.

Table S2. Pearson correlation for physiochemical and biological variables over study period (May - August 2018). Values in bold with an asterisk are significant at $\alpha = 0.05$.

	**Temp**	**Salinity**	**NO_3**	**NH_4**	**PO_4**	**Si**	**N:P**	**N:Si**	**Si:P**	**Chla**	**pDA**	**PN cells**
**Temp**	**1**	**−0.608***	0.0105	0.113	−0.174	−0.0116	0.174	−0.00731	0.0954	**−0.562***	**−0.558***	−0.348
	**Salinity**	**1**	0.0935	**−0.341***	0.396	−0.0609	**−0.489***	−0.129	−0.283	0.271	**0.574***	0.164
		**NO_3**	**1**	−0.0475	**0.513***	0.0225	−0.0590	0.121	−0.164	0.209	−0.0545	0.0788
			**NH_4**	**1**	−0.215	−0.0749	**0.918***	**0.794***	0.0499	0.190	−0.373	0.335
				**PO_4**	**1**	−0.0561	**−0.511***	−0.0249	**−0.489***	0.154	−0.0233	−0.0320
					**Si**	**1**	−0.0160	**−0.610***	**0.878***	−0.270	0.0415	−0.140
						**N:P**	**1**	**0.681***	0.256	0.156	−0.319	0.322
							**N:Si**	**1**	**−0.499***	0.431	−0.278	**0.459***
								**Si:P**	**1**	−0.301	0.0207	−0.120
									**Chla**	**1**	0.111	**0.863***
										**pDA**	**1**	−0.0111
											**PN Cells**	**1**
